# Oxidatively Damaged DNA in Rats Exposed by Oral Gavage to C_60_ Fullerenes and Single-Walled Carbon Nanotubes

**DOI:** 10.1289/ehp.11922

**Published:** 2008-11-12

**Authors:** Janne K. Folkmann, Lotte Risom, Nicklas R. Jacobsen, Håkan Wallin, Steffen Loft, Peter Møller

**Affiliations:** 1 Institute of Public Health, Department of Environmental Health, University of Copenhagen, Copenhagen, Denmark;; 2 National Research Centre for the Working Environment, Copenhagen, Denmark

**Keywords:** cancer, DNA damage, DNA repair, nanoparticle, oxidative stress

## Abstract

**Background:**

C_60_ fullerenes and single-walled carbon nanotubes (SWCNT) are projected to be used in medicine and consumer products with potential human exposure. The hazardous effects of these particles are expected to involve oxidative stress with generation of oxidatively damaged DNA that might be the initiating event in the development of cancer.

**Objective:**

In this study we investigated the effect of a single oral administration of C_60_ fullerenes and SWCNT.

**Methods:**

We measured the level of oxidative damage to DNA as the premutagenic 8-oxo-7,8-dihydro-2′-deoxyguanosine (8-oxodG) in the colon mucosa, liver, and lung of rats after intragastric administration of pristine C_60_ fullerenes or SWCNT (0.064 or 0.64 mg/kg body weight) suspended in saline solution or corn oil. We investigated the regulation of DNA repair systems toward 8-oxodG in liver and lung tissue.

**Results:**

Both doses of SWCNT increased the levels of 8-oxodG in liver and lung. Administration of C_60_ fullerenes increased the hepatic level of 8-oxodG, whereas only the high dose generated 8-oxodG in the lung. We detected no effects on 8-oxodG in colon mucosa. Suspension of particles in saline solution or corn oil yielded a similar extent of genotoxicity, whereas corn oil per se generated more genotoxicity than the particles. Although there was increased mRNA expression of 8-oxoguanine DNA glycosylase in the liver of C_60_ fullerene-treated rats, we found no significant increase in repair activity.

**Conclusions:**

Oral exposure to low doses of C_60_ fullerenes and SWCNT is associated with elevated levels of 8-oxodG in the liver and lung, which is likely to be caused by a direct genotoxic ability rather than an inhibition of the DNA repair system.

Humans have been exposed to particulate matter during much of evolution, but technologic achievements such as combustion engines and nanotechnology have yielded unique types of particles. Engineered nanotechnologic materials are projected to be used, for instance, in electronics, cosmetics, cleaning materials, coatings, food packaging, and medicines, with increasing human exposure. In addition, consumer products containing nanomaterials will inevitably end up as waste, and subsequent processing or deposition may liberate particles to the environment, where they might accumulate along the food chain because some are highly persistent (Helland et al. 2007). Reinforced attention to the hazardous properties of engineered nanoparticles has been evoked by a report showing that a single intra peritoneal application of carbon nanotubes generated mesotheliomas in p53^+/−^ mice (Takagi et al. 2008). These data are supported by another recent report showing that a single injection of carbon nanotubes into the peritoneal cavity of mice elicited an inflammatory reaction and granulomas at the peritoneal surface of the diaphragm (Poland et al. 2008). Similarly, pulmonary exposure to single-walled carbon nanotubes (SWCNT) produced granulomas in the lung of rodents (Lam et al. 2004; Shvedova et al. 2005; Warheit et al. 2004). It is possible that such nanotubes possess the same hazardous effects as other fibrous materials such as asbestos. On the other hand, particles such as C_60_ fullerenes might be less hazardous (Nielsen et al. 2008). For example, in a recent inhalation experiment, Baker et al. (2008) found that exposure to C_60_ fullerenes resulted in little pulmonary toxicity.

Generation of reactive oxygen species (ROS) and oxidative stress is generally accepted to be an important mechanism of action of nanoparticles (Ayres et al. 2008; Nel et al. 2006; Oberdörster et al. 2005). Exposure to combustion particles in urban air or diesel exhaust particles (DEP) is associated with oxidative stress, generated by particles themselves or by cell-mediated inflammatory responses and increased formation of oxidatively damaged DNA (Knaapen et al. 2004; Risom et al. 2005). 8-Oxo-7,8-dihydro-2′**-**deoxyguanosine-(8-oxodG) is considered to be an important oxidatively generated DNA lesion in carcinogenesis because *a*) it is mutagenic; *b*) mammalian cells have a highly versatile repair system for its removal; and *c*) the level of 8-oxodG is elevated in several types of tumor tissue (Evans et al. 2004). In addition, high urinary excretion of 8-oxodG is associated with increased risk of lung cancer among nonsmokers (Loft et al. 2006; Loft and Møller 2006). The relevance of 8-oxodG is further strengthened by observations that this lesion is elevated in animal tissues and human blood cells upon exposure to urban air pollution, diesel exhaust, or DEP (Møller et al. 2008). In particular, a single oral dose of DEP was associated with increased levels of 8-oxodG in liver, lung, and colon tissue (Danielsen et al. 2008b). Effect modification by the DNA repair system is likely to occur by continuous exposure. For example, ingestion of DEP in the diet for 3 weeks led to an up-regulation of the DNA repair system and unaltered levels of 8-oxodG in the liver and colon mucosa cells (Dybdahl et al. 2003), whereas measurements of lung tissue indicated unaltered regulation of the DNA repair system and elevated levels of oxidatively damaged DNA (Müller et al. 2004).

The aim of the present study was to investigate the effect of a single oral administration of C_60_ fullerenes and SWCNT. C_60_ fullerenes consist of 60 carbon atoms arranged in an aromatic soccer-ball structure with a nanosized diameter. SWCNT consists of two dimensions < 100 nm, whereas the axial dimension is much larger. Both C_60_ fullerenes and SWCNT are hydrophobic and hence difficult to suspend in saline solution, but they suspend more easily in oils. Consequently, rats received the particles in either saline solution or corn oil by oral gavage. We then assessed the level of 8-oxodG, a highly validated biomarker [European Standards Committee on Oxidative DNA Damage (ESCODD) et al. 2005]. We investigated alterations in the regulation of DNA repair by mRNA levels of 8-oxoguanine DNA glycosylase (*OGG1*), nei endonuclease VIII-like 1 (*E. coli*) (*NEIL1)*, mutY homolog (*E. coli*) (*MUTYH),* and nudix (nucleoside diphosphate linked moiety X)-type motif 1 (*NUDT1)*, which are involved in the removal of 8-oxodG in DNA and the nucleotide pool and may be up-regulated by oxidative stress (Hah et al. 2007; Risom et al. 2003a, 2007). We assessed the expression of heme oxygenase 1 (*HO1*) mRNA as a general marker of oxidative stress elicited by exposure to particulate matter (Ayres et al. 2008).

## Materials and Methods

### Particle exposure of animals

We obtained 84 female Fisher 344 rats from Taconic (Ry, Denmark). Animals were acclimatized for at least 1 week before entering the experiments. The rats were housed in a temperature- controlled (22–24°C) and moisture-controlled (40–70%) room with a 12-hr light/12-hr dark cycle. All animals had free access to tap water and Standard Altromin no. 1314 rat chow (Altromin, Lage, Germany) during the acclimation and housing/treatment periods. Rats were sacrificed at 9 weeks of age. Animals were treated humanely and with regard for alleviation of suffering. All animal procedures followed the guidelines for the care and handling of laboratory animals established by the Danish government, and the Animal Experiment Inspectorate, Ministry of Justice, approved the study (no. 2006/561-1161).

The dry powder of C_60_ fullerenes was described by the manufacturer to be a 99.9% pure preparation with a primary particle size of 0.7 nm (Sigma-Aldrich, Brøndby, Denmark). The dry powder of SWCNT was described by the manufacturer to have a primary particle size of 0.9–1.7 nm and a fiber length < 1 μm (Thomas Swan and Co Ltd, Consett, UK). We suspended the particles in either saline or corn oil (Sigma-Aldrich) by sonication at 70 W and 42 kHz (Branson 1510, VWR–Bie & Berntsen A/S, Herlev, Denmark) in a 5-day period for 10 hr each day and again 30 min before administration. For C_60_ fullerene and SWCNT, the gas exchange surface areas were < 20 m^2^/g and 731 ± 2 m^2^/g and the average pore sizes were 0 and 15 nm respectively (Jacobsen et al. 2008b). We used dynamic light scattering, as described by Jacobsen et al. (2008b), to measure the particle size in suspensions at the same concentration as administered to the rats by oral gavage. In general, it was difficult to determine the presence of nanoparticles in both types of solutions by dynamic light scattering because the solutions contain agglomerates with larger particle sizes. The particle sizes of C_60_ fullerenes in the saline solution were 407 nm in the low dose and 621 and 5,117 nm in the high dose. In saline solution, the particle sizes of SWCNT were 195, 797, and 5,457 nm (low dose); the particle size in the highest dose could not be determined by dynamic light scattering. The particles were easier to suspend in corn oil than in saline. We obtained size modes of the C_60_ fullerenes in corn oil by dynamic light scattering as follows: 234 nm (low dose); 40, 713, and 3,124 nm (high dose). The SWCNT had size modes as follows: 34 and 178 nm (low dose) and 1,015 nm (high dose). The SWCNT contained transition metals (2% iron and traces of cobalt, nickel, and manganese) and polycyclic aromatic hydrocarbons [PAHs; 417 ng/g of the U.S. Environmental Protection Agency (EPA) priority PAH compounds (U.S. EPA 1991)], whereas neither transition metals nor PAH could be detected in C_60_ fullerenes.

The rats received a single intragastric dose of the particle preparations by oral gavage (0.064 and 0.64 mg/kg body weight suspended in saline or corn oil; *n* = 8), saline solution (control; *n* = 10), or corn oil (control; *n* = 10). Each rat received 200 μL fluid. The rats were sacrificed by cervical dislocation 24 hr after intragastric administration. The liver, lung, and colon tissues were snap-frozen in liquid nitrogen and stored at −80°C. We investigated effects in these organs because they are along the likely local and systemic exposure route and we have data from similar experiments on DEP as Standard Reference Material 2975 (SRM2975; National Institute of Standards and Technology, Gaithersburg, MD, USA), which was previously reported to be associated with increased levels of DNA damage 24 hr after a single intragastric application (Danielsen et al. 2008b). For all experiments, rats from each group (0, 0.064, and 0.64 mg particles/kg body weight) were treated the same day.

### ROS-generating ability

The ability of the C_60_ fullerenes, SWCNT, SRM2975, and Printex 90 carbon black (Degussa-Hüls, Frankfurt, Germany) to generate ROS in aqueous solution was determined by oxidation of 2′,7′-dichloro dihydrofluorescin (Molecular Probes, Portland, OR, USA). The particle suspensions were prepared in Hank’s balanced saline solution and sonicated immediately before incubation, as described previously by Jacobsen et al. (2008b). The oxidation product (2′,7′-dichlorofluorescein) was determined by fluorescence spectrometry with excitation at 490 nm and emission at 520 nm on a fluorescence spectrophotometer (Victor Wallac-2 1420; PerkinElmer, Hvidovre, Denmark).

### Oxidatively damaged DNA

We obtained suspensions of colon epithelial cells by scraping off the cells on the luminal side of the colon with a glass slide in ice-cold Merchant buffer, as described previously by Dybdahl et al. (2003). The DNA was extracted from colon mucosa cells, liver, and lung tissue according to the procedure recommended by ESCODD et al. (2005). We isolated nuclei in buffer containing deferoxamine mesylate (Sigma-Aldrich) to prevent spurious oxidation; this was followed by lysis, RNase, and proteinase treatment (Sigma-Aldrich). We extracted DNA in buffer containing 40 mM Tris, 20 mM Na_2_EDTA, 7.6 M NaI, and 0.3 mM deferoxamine mesylate, pH 8.0 (Sigma-Aldrich), 2-propanol, and ethanol. We digested DNA extracts to nucleosides using nuclease P1 and alkaline phosphatase (Merck, Darmstadt, Germany) and measured 8-oxodG and dG by HPLC with electrochemical and ultraviolet detection, respectively.

### mRNA expression of HO1, MUTYH, NEIL1, NUDT1, and OGG1

We purified total RNA from liver and lung tissue using TRIzol Reagent (Invitrogen, San Diego, CA, USA) and DNase treatment according to the manufacturer’s protocol (SV Total RNA isolation kit; Promega, Madison, WI, USA). For cDNA synthesis, 100–200 ng RNA was used in a reaction volume of 20 μL using GeneAmp RNA PCR (polymerase chain reaction) Kit (Applied Biosystems, Nærum, Denmark) as recommended by the manufacturer.

We analyzed mRNA levels of the following genes: *HO1* [GeneID 24451 (National Center for Biotechnology Information 2008)], *MUTYH* (GeneID 170841), *NEIL1* (GeneID 367090), *NUDT1* (GeneID 117260), *OGG1* (GeneID 81528) on a Taqman ABI7900 (Applied Biosystems) as described by Risom et al. (2003b). We used Taqman probes and primers *MUTYH* (Rn00591196), *NEIL1* (Rn01422330), *NUDT1* (Rn00589097), and Taqman 18S probe (Euk 18S rRNA FAM/MGB probemix) from Applied Biosystems. All probes and primers span exon junctions and are cDNA specific. We used oligonucleotides of *HO1* and *OGG1* as follows (final concentrations are shown in parentheses):

- *RnHO1* forward primer (900 nM), 10F: 5′-CCA CAG CTC GAC AGC ATG T-3′; reverse primer (900 nM), 159R: 5′-GGA GGC CAT CAC CAG CTT AAA-3′; Taqman probe (250 nM), 135T: 5′6-FAM-TTC CCT GGA CAC CTG ACC CTT CTG A-TAMRA-3′
- *RnOGG1* forward primer (900 nM), 401F: 5′-TGG CTC AGA AAT TCC AAG GTG T-3′; reverse primer (900 nM) 609R, 5′-TAC TTC TGG ACC AGC CAG GG-3′; Taqman probe (250 nM), 468T: 5′6-FAM-CTG TTC TTC CAA CAA CAA CAT TGC TCG C-TAMRA-3′.

For the PCR reactions, we mixed 4 μL of the cDNA preparation with master mix and water to a final volume of 228 μL. Aliquots were mixed with probes and primers to a final volume of 36 μL; and then transferred to three wells at 10 μL/well. For the PCR reaction, we used the following protocol: activation of the polymerase, 95°C for 20 sec; followed by 45 cycles of 95°C for 1 sec; and finally 60°C for 20 sec. We measured the expressions of reference (18S) and target genes in separate tubes. The expression levels are reported as target mRNA normalized to 18S, calculated by the comparative cycle threshold (C_t_) method 2^−Δ C_t_^. The SD of the triplicates was < 0.5 (based on C_t_ values). Each run included a standard (for verification of the PCR efficiency) and negative (no template) and positive controls.

### OGG1 repair activity

We obtained extracts of liver tissue by using a plunger to force the tissue through a sieve in one end of a stainless steel cylinder (0.5 cm in diameter, mesh size 0.4 mm) while the cylinder was submerged in 2 mL ice-cold buffer (45 mM HEPES, 0.4 M KCl, 1 mM EDTA, 0.1 mM DTT, 10% glycerol, pH 7.8), as described previously (Folkmann et al. 2007). The protein concentrations of the liver tissue extracts were measured using the Coomassie Plus – The Better Bradford Assay Reagent as recommended by the manufacturer (Pierce, Rockford, IL, USA) using bovine serum albumin as standard. We diluted liver tissue extracts to a final protein concentration of 3 mg/mL.

We analyzed *OGG1* repair activity as the number of incisions in substrate nuclei (Guarnieri et al. 2008). We prepared substrate nuclei containing 8-oxodG by treating THP-1 human monocyte cells (American Type Culture Collection, Manassas, VA, USA) with 1 μM Ro19-8022 in phosphate-buffered saline and irradiating them with white light for 4 min. The Ro19-8022 photosensitizer was a gift from F. Hoffmann-La Roche Ltd (Basel, Switzerland). Substrate nuclei were embedded in low-melting-point gel (0.75%; Sigma-Aldrich), applied onto GelBond films (Cambrex; Medinova Scientific A/S, Hellerup, Denmark), and lysed in ice-cold lysis solution (2.5 M NaCl, 0.1 mM Na_2_EDTA, 10 mM Tris, 1% Triton X-100, pH 10) for 1 hr at 4°C. We applied liver tissue extracts onto the gels and incubated them for 20 min at 37°C in a humid box. Negative controls (buffer) and positive controls (formamidopyrimidine DNA glycosylase; gift from A. Collins, University of Oslo, Oslo, Norway) were incubated for 45 min.

The GelBonds were subsequently treated in ice-cold alkaline solution (300 mM NaOH, 1 mM Na_2_EDTA, pH > 13.0) for 40 min and electrophoresed 20 min in the same solution at 25 V (0.83 V/cm) and 300 mA. The nuclei were visualized using fluorescence microscope (Olympus, Ballerup, Denmark) at 40× magnification after staining with the YOYO-1 fluorescent dye (Molecular Probes). We scored the level of DNA damage according to five classes of damage of 100 randomly selected nuclei from each sample; we examined two slides for each sample. The samples were coded before scoring. We determined the repair activity of the tissue extracts as the difference in score (arbitrary units) between parallel gels incubated with extract and control solution, respectively.

### Statistics

We analyzed the results statistically by two-factor analysis of variance (ANOVA) using the dose of particles and vehicle as categorical variables. We found no interactions between the categorical factors; thus, the *p-*values correspond to single -factor effects. We tested the homogeneity of variance by Levene’s test. To achieve homogeneity of variance, we log_10_-transformed all results, except those for 8-oxodG in the lung. Statistically significant effects are reported as the percent increase with either 95% confidence intervals (CIs) or *p*-values of post hoc Fisher least statistical difference tests. The statistical analysis was carried out using Statistica for Windows Version 5.5 (StatSoft, Inc., Tulsa, OK, USA).

## Results

### ROS-generating ability

**Figure 1:**
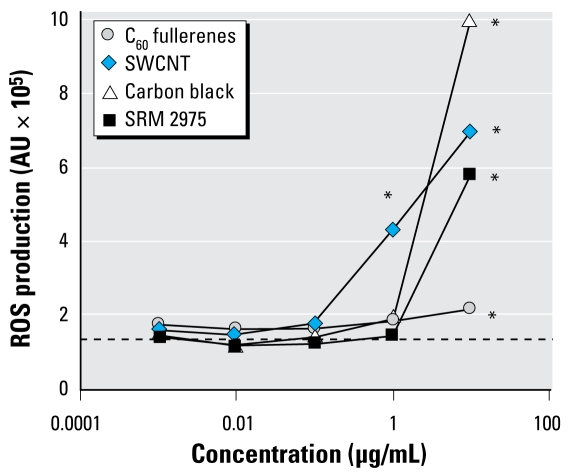
ROS production [in arbitrary units (AU)] by C_60_ fullerenes, SWCNT, Printex 90 carbon black, and SRM2975 in Hank’s balanced saline solution detected as 2′,7′-dichlorofluorescein. Each point represents the mean ROS production of duplicate replicates in two independent experiments; the dotted line represents incubations without particles (quadruplicate in two independent experiments). ^*^p < 0.05 compared with incubations without particles.

Figure 1 shows ROS generation of particles in aqueous solution. The ROS generation was 3.3-fold (95% CI, 2.1–4.5) and 5.5-fold (95% CI, 4.2–6.6) in incubations containing SWCNT at 1 and 10 μg/mL, respectively. The C_60_ fullerenes increased ROS production at the highest dose by 1.6-fold (95% CI, 1.5–1.9). SRM2975 and carbon black increased the level of ROS production by 4.4-fold (95% CI, 4.3–4.5) and 7.6-fold (95% CI, 7.4–7.9), respectively, at 10 μg/mL (Figure 1).

### Oxidatively damaged DNA

**Figure 2:**
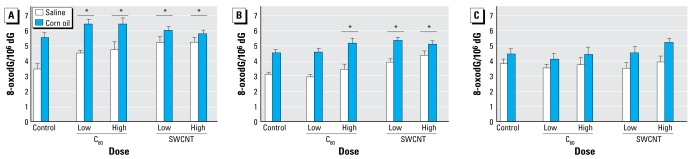
Level of 8-oxodG in liver (*A*), lung (*B*), and colon (*C*) tissue 24 hr after oral exposure to a single dose of C_60_ fullerenes or SWCNT suspended in saline or corn oil. Values shown are mean ± SE of 8 treated animals and 10 control animals. ^*^p < 0.05 compared with the control group (single-factor effect of particle exposure, ANOVA).

Figure 2 shows the level of 8-oxodG in colon, liver, and lung tissue of rats administered C_60_ fullerenes and SWCNT dispersed in either saline or corn oil. We observed significant single-factor effects of corn oil, which was associated with 25% (95% CI, 12–40), 30% (95% CI, 20–40), and 38% (95% CI, 29–47) higher level of 8-oxodG in the colon, liver, and lung, respectively.

We found no interactions between type of vehicle and particle exposure, indicating that particles in corn oil generated the same level of DNA damage as particles in saline. The generation of 8-oxodG is based on data from both types of vehicles (corresponding to estimates of single-factor effects). In the liver, the exposure to C_60_ fullerenes was associated with 17% (95% CI, 4–34) and 25% (95% CI, 11–41) increases of 8-oxodG in groups treated with the low dose and high dose of particles, respectively. Exposure to SWCNT increased the level of 8-oxodG in the liver by 22% (95% CI, 8–38) and 20% (95% CI, 7–36) in the rats given the low dose and high dose, respectively. There were increased levels of 8-oxodG in the lung after exposure to SWCNT at both the low [21% (95% CI, 9–33)] and high doses [23% (95% CI, 11–35)], whereas only the high dose of C_60_ fullerenes was associated with significantly elevated levels of 8-oxodG in the lung [18% (95% CI, 4–31)]. Oral exposure to particles was not associated with increased levels of 8-oxodG in colon mucosa tissue.

### *mRNA expression of* HO1, MUTYH, NEIL1, NUDT1, *and* OGG1

Table 1 outlines the gene expression of *OGG1, HO1, NEIL1, MUTYH*, and *NUDT1* mRNA in liver and lung tissue. The type of vehicle did not affect the level of gene transcription (*p* > 0.05, single-factor effect in ANOVA). The low and high doses of C_60_ fullerenes increased the gene expression of *OGG1* by 1.30-fold (95% CI, 0.9–1.9) and 1.80-fold (95% CI, 1.2–2.6), respectively, in the liver, whereas a similar effect of SWCNT did not reach statistical significance. In contrast, we found no significant effects in the expression of *HO1, NEIL1, MUTYH*, and *NUDT1* mRNA in liver and lung tissue. Colon tissue was not analyzed because there was unaltered level of 8-oxodG and limited amounts of material were available.

### OGG1 repair activity

**Figure 3:**
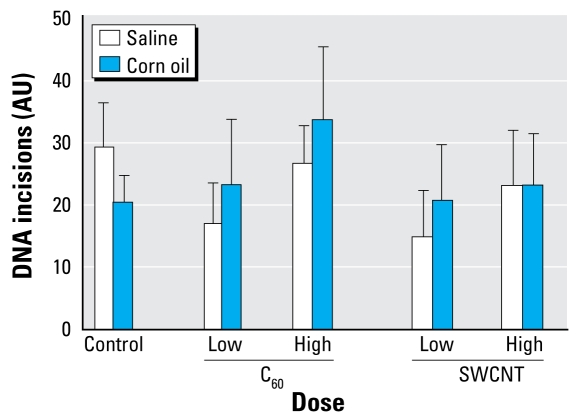
*OGG1* repair activity in the liver 24 hr after exposure to a single dose of C_60_ fullerenes or SWCNT dissolved in saline or corn oil. The activity is indicated as the number of repair incisions in arbitrary units (AU) of substrate nuclei treated with Ro19-8022 and white light, which generate oxidative damage to the DNA. Values shown are mean ± SE for 8 treated animals and 10 control animals).

Based on the observation that the gene expression of *OGG1* was increased in the liver after oral exposure to particles, we also measured the *OGG1* repair activity of liver extracts (Figure 3). Neither the exposure to particles nor the vehicle were associated with a significantly altered level of *OGG1* repair activity, although a dose-related effect of C_60_ fullerenes is suggested (*p* = 0.20, ANOVA).

## Discussion

In this study we found increased levels of oxidatively damaged DNA in liver and lung tissue 24 hr after oral administration of C_60_ fullerenes and SWCNT in saline or corn oil. There were virtually no alterations of the repair activity of oxidized base lesions in the same tissues, indicating that the level of DNA damage is not underestimated as a consequence of increased repair.

The levels of 8-oxodG were approximately 20% increased in the liver and lung tissue after the exposure to C_60_ fullerenes and SWCNT, whereas the vehicle did not affect the particle-generated genotoxicity. The effect of C_60_ fullerenes on *OGG1* mRNA expression in the liver and 8-oxodG in the lung showed clear dose–response relationships, whereas other dose–response relationships were more flat. Possible agglomeration of particles affecting uptake and effects might have influenced the dose relationships. This could also be relevant for the lack of genotoxicity in colon mucosa cells. These cells have high turnover, which could dilute any possibly transient DNA damage, whereas liver and lung cells have a low proliferation rate, allowing damage to accumulate. We have previously shown that rats given the same oral dose of DEP as SRM2975 had approximately 50% elevated level of 8-oxodG in colon mucosa cells, liver, and lung (Danielsen et al. 2008b). Thus, our results indicate that C_60_ fullerenes and SWCNT are genotoxic in rats, but the effect is lower than that observed for SRM2975. The C_60_ fullerenes and SWCNT were administered in rather low doses because we wanted to use the SRM2975 preparation as benchmark particles. In comparison, a recent study with intravenous administration of 150 mg pegylated SWCNT per mouse argued that concentrations between 10- and 100-fold lower were sufficient in biomedical applications (Schipper et al. 2008). Other recent studies on pulmonary toxicity of SWCNT have used doses in the range of 20–40 μg/mouse (corresponding to about 1–2 mg/kg body weight) by pharyngeal aspiration (Han et al. 2008; Shvedova et al. 2008). The doses we used in the present study can thus be considered as being in the lower range of exposures.

To the best of our knowledge, our data are the first to demonstrate that C_60_ fullerenes and SWCNT generate oxidatively damaged DNA in rodent organs. Other reports have shown that C_60_ fullerenes and SWCNT exposure of cells in culture is associated with elevated levels of DNA damage measured by the comet assay (Dhawan et al. 2006; Kisin et al. 2007; Pacurari et al. 2008). These types of lesions represent general genotoxicity rather than oxidatively damaged DNA, which can be assessed by a modified version of the comet assay including digestion with DNA glycosylase or endonuclease enzymes (Møller 2006). We used this approach in a cell culture experiment and demonstrated that both C_60_ fullerenes and SWCNT generated oxidatively damaged DNA (Jacobsen et al. 2008b). The oxidizing effect of C_60_ fullerenes on biomolecules in cell culture settings could be due to generation of singlet oxygen induced by photosensitization (Kamat et al. 2000; Yamakoshi et al. 2003). Oxidations of DNA in internal organs probably arise from mixed ROS generation, as supported by increased mortality after C_60_ fullerene exposure in zebrafish in the dark and by enhanced mortality resulting from coexposure of C_60_ fullerenes with hydrogen peroxide or glutathione depletion (Usenko et al. 2008). Hydroxyl radicals appear to be the important type of ROS generated by SWCNT in cell cultures (Manna et al. 2005; Pacurari et al. 2008). The level of transition metals is not likely to explain the differences in genotoxicity between the particles used in the present study and SRM2975, because the latter has very low levels, whereas the SWCNT used here contained 2% iron, as well as traces of other metals, and the C_60_ fullerenes had undetectable levels. Moreover, SWCNT at low concentrations produced higher levels of ROS in cell-free systems than did SRM2975 and C_60_ fullerenes, whereas SRM2975 appeared to induce more guanine oxidation than the engineered particles in cell culture (Danielsen et al. 2008a; Jacobsen et al. 2008a, 2008b). The SRM2975 preparation contains substantial amounts of PAHs, which show low levels in SWCNT and are undetectable in C_60_ fullerenes. The content of PAHs could explain the higher DEP-induced level of 8-oxodG possibly through generation of oxidative stress by the aldo-ketoreductase metabolic pathway, as recently shown in cell culture (Park et al. 2008). Nevertheless, particles such as carbon black with very low PAH and metal levels induced the highest levels of ROS in both cell-free systems and in cell culture with resulting DNA oxidation and mutagenicity (Jacobsen et al. 2007), indicating that particles as such have the ability to induce oxidative stress. Accordingly, ROS generation in an acellular *in vitro* system does not appear to predict the ability of particles to oxidatively damage DNA in cell culture or *in vivo*.

The notion that C_60_ fullerenes and SWCNT induce less oxidative stress compared with DEP is supported by the unaltered regulation of *HO1*, which in our previous study was up-regulated by exposure to SRM2975 (Danielsen et al. 2008b). In the present experiments, we observed unaltered expression levels of *NEIL1, MUTYH*, and *NUDT1*, whereas the increased expression of *OGG1* mRNA in the liver of rats exposed to C_60_ fullerenes was not associated with higher repair activity, which might be due to the fact that longer exposure times are required to observe increased repair activity. Indirect evidence of this effect modification comes from studies of inhalation exposure to DEP where repeated exposures on 4 consecutive days were associated with up-regulation of *OGG1* and unaltered 8-oxodG in pulmonary tissue (Risom et al. 2003a), whereas an identical exposure scenario yielded higher levels of 8-oxodG in *OGG1* knockout mice (Risom et al. 2007). Although increased expression levels of *OGG1* might be considered beneficial to the cells, it should be emphasized that in human beings, with the variant form of *OGG1* (Ser326Cys) genotype, high expression levels of *OGG1* mRNA in leukocytes is associated with increased risk of lung cancer (Hatt et al. 2008).

Although our results strongly indicate that oral exposure to C_60_ fullerenes and SWCNT is associated with increased generation of oxidized DNA, it is still unresolved how application of particulate matter in the gut can oxidize biomolecules in internal organs. A somewhat naive notion suggests that the epithelial lining of the gut is designed to absorb substances and thus may allow passage of particulate matter. In fact, we suspended the particles in either corn oil or saline solution because of the notion that their hydrophobic nature would let them follow the regular passage of lipids in the gut. Because there was no difference in genotoxicity between the particles suspended in corn oil and saline solution, we believe that orally administered particles will distribute in the chyle of the intestinal juice, despite the fact that they may reach the gastrointestinal tract as agglomerates, as indicated by aqueous particle suspension characterization. The results suggest that the particles are absorbed from the gastrointestinal tract to blood circulation and secondary organs. However, at present the extent of translocation of particulate matter across epithelial barriers is a highly controversial issue. Studies of the pulmonary translocation of model particles to systemic circulation and secondary organs indicate only minute passage (Kreyling et al. 2002; Mills et al. 2006; Möller et al. 2008; Wiebert et al. 2006). On the other hand, whole-body inhalation exposure to ultrafine carbon particles have suggested some deposition in the liver, which has been speculated to originate from gastro intestinal exposure and uptake from the gut (Oberdörster et al. 2002). In addition, the uptake of C_60_ fullerenes and polystyrene latex microspheres from the gastrointestinal tract into blood circulation has been estimated in the range of 1% of the applied dose (Carr et al. 1996; Yamago et al. 1995). However, it is interesting to note that in drug delivery research, there is acceptance that particulate matter can be absorbed from the gastrointestinal tract, and this feature of nanomaterials is being used as an approach of altering the pharmacokinetic behavior of drugs (Florence 2004).

We observed an effect of corn oil in all three organs. This was not part of our *a priori* hypothesis. We chose the volume of corn oil to be identical to that of the saline solution (200 μL), which would allow a reasonable volume for the suspension in the aqueous solution. It should be emphasized that corn oil is rich in polyunsaturated fatty acids and was sonicated, which might produce genotoxic compounds. In addition, rat studies indicated that a diet rich in corn oil increased 8-oxodG excretion in urine (Loft et al. 1998) as well as the levels of 5-hydroxymethyl-2′-deoxyuridine, another marker of oxidatively damaged DNA in blood and mammary gland tissue (Djuric et al. 2001). A dyslipidemic state, as found in apoE knockout mice, also appears to include age-dependent accumulated oxidized DNA base lesions in the liver (Folkmann et al. 2007).

In conclusion, the data obtained from the present study indicate that C_60_ fullerenes and SWCNT generated oxidatively damaged DNA in liver and lung cells by a gastro intestinal route. The genotoxic effect resulting from exposure to C_60_ fullerenes and SWCNT was smaller than that from DEP exposure; however, it may be a cause for concern in humans.

## Figures and Tables

**Table 1:** mRNA expression levels of genes involved in the removal of oxidized DNA base lesions in the liver and lung 24 hr after oral administration of C_60_ fullerenes or SWCNT suspended in either saline or corn oil.

	Particle dose in saline (mg/kg body weight)			Particle dose in corn oil (mg/kg body weight)		
Tissue	0	0.064	0.64	0	0.064	0.64
C_60_ fullerenes						

Lung						
*HO1*	10.4 ± 1.70	6.30 ± 0.76	6.26 ± 0.96	5.79 ± 0.58	8.63 ± 2.41	7.66 ± 1.50
*MUTYH*	6.26 ± 1.23	2.97 ± 0.56	4.75 ± 0.95	5.65 ± 1.50	7.71 ± 1.67	8.94 ± 3.46
*NEIL1*	3.16 ± 0.49	2.26 ± 0.51	2.01 ± 0.33	2.94 ± 0.862	2.97 ± 0.87	3.53 ± 1.01
*NUDT1*	0.34 ± 0.04	0.27 ± 0.02	0.40 ± 0.09	1.26 ± 0.75	1.37 ± 0.49	1.29 ± 0.97
*OGG1*	0.55 ± 0.07	0.34 ± 0.06	0.38 ± 0.05	0.49 ± 0.07	0.57 ± 0.11	0.57 ± 0.10
Liver						
*HO1*	1.05 ± 0.12	1.55 ± 0.28	1.32 ± 0.18	1.47 ± 0.28	1.49 ± 0.37	1.27 ± 0.22
*MUTYH*	0.36 ± 0.07	0.45 ± 0.09	0.69 ± 0.30	0.35 ± 0.04	0.34 ± 0.05	0.40 ± 0.06
*NEIL1*	0.86 ± 0.16	1.08 ± 0.25	1.57 ± 0.74	0.72 ± 0.10	0.58 ± 0.13	0.70 ± 0.08
*NUDT1*	0.25 ± 0.07	0.35 ± 0.1	0.47 ± 0.18	0.26 ± 0.09	0.18 ± 0.03	0.21 ± 0.07
*OGG1*	0.12 ± 0.02	0.14 ± 0.02	0.25 ± 0.07*	0.10 ± 0.02	0.14 ± 0.03	0.16 ± 0.03*

SWCNT						

Lung						
*HO1*	10.4 ± 1.70	5.03 ± 1.04	14.1 ± 7.81	5.79 ± 0.58	7.15 ± 1.73	8.14 ± 2.79
*MUTYH*	6.26 ± 1.23	3.48 ± 1.07	9.80 ± 3.54	5.65 ± 1.50	8.65 ± 3.23	3.63 ± 0.68
*NEIL1*	3.16 ± 0.49	2.03 ± 0.56	2.80 ± 0.75	2.94 ± 0.862	4.88 ± 1.45	2.24 ± 0.53
*NUDT1*	0.34 ± 0.04	0.32 ± 0.08	1.78 ± 0.81	1.26 ± 0.75	2.29 ± 1.15	0.47 ± 0.12
*OGG1*	0.55 ± 0.07	0.34 ± 0.03	1.17 ± 0.59	0.49 ± 0.07	0.54 ± 0.10	0.45 ± 0.06
Liver						
*HO1*	1.05 ± 0.12	1.56 ± 0.23	1.65 ± 0.19	1.47 ± 0.28	1.05 ± 0.14	1.30 ± 0.22
*MUTYH*	0.36 ± 0.07	0.36 ± 0.05	0.51 ± 0.09	0.35 ± 0.04	0.37 ± 0.05	0.30 ± 0.06
*NEIL1*	0.86 ± 0.16	0.88 ± 0.15	0.83 ± 0.17	0.72 ± 0.10	0.69 ± 0.11	0.57 ± 0.09
*NUDT1*	0.25 ± 0.07	0.22 ± 0.03	0.48 ± 0.20	0.26 ± 0.09	0.37 ± 0.12	0.26 ± 0.05
*OGG1*	0.12 ± 0.02	0.15 ± 0.03	0.16 ± 0.04	0.10 ± 0.02	0.15 ± 0.04	0.17 ± 0.04

The mRNA expression is relative to the expression of 18S per 106. Values are mean ± SE.

* *p* < 0.05 (single-factor effect of the particle exposure, ANOVA).
